# Brain-Gut Interplay: Cognitive Performance and Biomarker Correlations in IBD Patients

**DOI:** 10.3390/jcm14072293

**Published:** 2025-03-27

**Authors:** Oliviu-Florențiu Sârb, Maria Iacobescu, Andreea-Maria Soporan, Ximena-Maria Mureșan, Adriana-Daniela Sârb, Raluca Stănciulescu, Corneliu-Daniel Leucuța, Alina-Ioana Tanțău

**Affiliations:** 1 Department of Neuroscience, Iuliu Hatieganu University of Medicine and Pharmacy, 400012 Cluj-Napoca, Romania; sarboliviu@yahoo.com; 2 Department of Internal Medicine, 4th Medical Clinic, Iuliu Hatieganu University of Medicine and Pharmacy, 400015 Cluj-Napoca, Romania; alitantau@gmail.com; 3 Personalized Medicine and Rare Diseases Department MEDFUTURE-Institute for Biomedical Research, Iuliu Hatieganu University of Medicine and Pharmacy, 400347 Cluj-Napoca, Romania; andreea.ungur@medfuture.ro (A.-M.S.); ximena.muresan@medfuture.ro (X.-M.M.); 4 Pharmaceutical Analysis, Faculty of Pharmacy, Iuliu Hatieganu University of Medicine and Pharmacy, 400006 Cluj-Napoca, Romania; 5 Department of Internal Medicine, Heart Institute, Iuliu Hatieganu University of Medicine and Pharmacy, 400001 Cluj-Napoca, Romania; adriana_daniela_porca@elearn.umfcluj.ro; 6 Department of Gastroenterology, Octavian Fodor Regional Institute of Gastroenterology and Hepatology, 400158 Cluj-Napoca, Romania; stanciulescu_raluca_maria@elearn.umfcluj.ro; 7 Department of Biostatistics and Informatics, Iuliu Hatieganu University of Medicine and Pharmacy, 400006 Cluj-Napoca, Romania; dleucuta@elearn.umfcluj.ro

**Keywords:** inflammatory bowel disease, crohn’s disease, ulcerative colitis, neurocognitive disease, dementia, gut-brain axis, depression, montreal cognitive assessment

## Abstract

**Background/Objectives:** Inflammatory bowel diseases (IBD), including mainly ulcerative colitis (UC) and Crohn’s disease (CD), have been associated with cognitive and psychological changes, though the mechanisms remain unclear. **Methods**: This prospective case-control study aimed to evaluate cognitive performance and biomarkers (homocysteine, serum amyloid A, brain-derived neurotrophic factor, and S100B protein) in IBD patients. **Results**: A total of 90 individuals (34 UC, 21 CD, and 35 controls) were assessed using the Montreal Cognitive Assessment (MoCA), the Memory Impairment Index (MIS), and biomarker analysis. MoCA and MIS testing showed significant differences between UC, CD, and the controls, with lower scores observed in IBD groups (*p* = 0.003, *p* = 0.015). Regarding trail-making tests, digit symbol substitution tests, and forward and backward digit spans, no significant changes were observed. No functional deficits were observed in daily activities. Biomarker analysis revealed lower brain-derived neurotrophic factor and higher serum amyloid A levels in IBD patients, correlated to MOCA and MIS scores. There were no significant differences in psychological distress between IBD patients and the controls. Subtle cognitive declines were noted across all groups during the 1-year follow-up, without any statistical significance when groups were compared. **Conclusions**: In conclusion, IBD patients reported lower cognitive scores compared to the controls, while no differences in depression and anxiety scores were observed. Higher BDNF levels correlated with better cognitive functioning, while higher serum amyloid A correlated with lower cognitive functioning.

## 1. Introduction

Inflammatory bowel disease (IBD) is a group of conditions characterized by chronic intestinal inflammation, with the most common types being Crohn’s disease (CD) and ulcerative colitis (UC) [1,2]. Although the exact mechanisms of IBD remain unclear, its pathogenesis is thought to involve a complex interplay of genetic predisposition, environmental triggers, and immune dysregulation. A hallmark of IBD is an abnormal immune response where the body mistakenly targets gut microbiota, leading to intestinal inflammation, disruption of the intestinal barrier, and systemic inflammation. This chronic inflammatory state perpetuates tissue damage and is central to the disease’s progression [3].

Mild cognitive impairment (MCI) is characterized by cognitive decline that exceeds normal aging but does not significantly interfere with daily functioning. Risk factors for MCI include aging, genetics, lifestyle behaviors, and medical conditions [4]. Emerging evidence suggests that systemic inflammation, including chronic inflammation seen in IBD, may contribute to cognitive impairment by promoting neuroinflammation and oxidative stress [5,6]. Notably, some studies have identified a potential association between IBD and an increased risk of Alzheimer’s disease (AD) or other neurodegenerative disorders, suggesting shared inflammatory pathways [5,7,8].

Several biomarkers, including homocysteine (Hcy), serum amyloid A (SAA), brain-derived neurotrophic factor (BDNF), and S100 calcium-binding protein B (S100B), have been implicated in both IBD and neurodegenerative processes [9]. Elevated Hcy levels in IBD patients have been associated with cerebrovascular damage, oxidative stress, and cognitive decline [10,11]. SAA, an acute-phase protein produced during inflammation, was found to contribute to neuroinflammation and neuronal damage, though its role in IBD is not well understood. Chronic gut inflammation in IBD has been linked to increased amyloid protein levels [12,13,14]. Similarly, BDNF, essential for neuronal growth and synaptic function, was reported to be dysregulated in IBD and linked to neurological disorders, though its exact role in IBD-associated inflammation remains unclear [15,16]. Lastly, S100B, a protein involved in neuroinflammation and oxidative stress, was found to be elevated in both the intestinal tissue and serum of IBD patients, reflecting damage to the enteric nervous system and the systemic effects of inflammation [17].

### Aim of the Study

The aim of this study was to explore the role of gut-brain axis (GBA) interactions in the development of MCI in patients diagnosed with IBD. The focus was on the effects of the disruption of GBA on the cerebral functions, mainly cognitive function and affective mood. Blood biomarkers associated with cognitive dysfunction, namely SAA, Hcy, S100B, and BDNF, were evaluated as potential predictors for the development of MCI or dementia in the study population.

## 2. Material and Methods

### 2.1. Study Design and Assessment Methodology

A comprehensive research protocol was developed and published at the outset of the study [18], ensuring a structured and transparent approach. This facilitated adherence to predefined methodologies, minimized the risk of bias, and enhanced the study’s reproducibility. Cognitive assessments were conducted by an examiner officially certified to administer the Montreal Cognitive Assessment (MoCA), ensuring the validity and reliability of the evaluations. The Memory Index Score (MIS), derived from the MoCA, was used to provide a focused measure of memory performance, offering a more nuanced analysis of memory impairment in the study population. Additional tools included the Depression, Anxiety, and Stress Scale (DASS-21), as well as the Activities of Daily Living (ADL) and Instrumental Activities of Daily Living (IADL) questionnaires, which were sourced from publicly available materials. Permission was obtained for the EQ-5D-5L questionnaire to assess quality of life, ensuring compliance with copyright and ethical guidelines [19,20]. Cognitive performance was further evaluated using the Trail Making Tests A and B (TMT-A and TMT-B), the Digit Symbol Substitution Test (DSST), and the Forward and Backward Digit Span (FDS and BDS) tests, all sourced from freely available resources. This methodology ensured the ethical and rigorous use of validated assessment tools while maintaining accessibility for data collection.

### 2.2. Design and Participants

We conducted a single-center observational, prospective, analytic case-control study from December 2021 to May 2024. The study focused on patients from medical clinics in Cluj-Napoca, including the Institute of Gastroenterology and Hepatology O. Fodor, the County Emergency Hospital, and the Clinical CF Hospital. Three separate groups consisting of IBD patients (CD group, UC group) and a control group (CG) were recruited in the study. A follow-up was performed at 50–54 weeks after the baseline visit.

### 2.3. Inclusion Criteria

Patients in the CD and UC groups were required to meet the following inclusion criteria: male or female, age over 18 years, a confirmed diagnosis of IBD, and signed informed consent. Patients in the IBD groups were required to meet specific remission criteria: CD patients needed to have a Crohn’s Disease Activity Index (CDAI) score below 150, while UC patients required a Harvey-Bradshaw Index (HBI) score below 2. Participants in the CG were required to be male or female, age over 18 years, with no diagnosis of IBD and a good level of overall health.

### 2.4. Exclusion Criteria

The absolute exclusion criteria were prior stroke or myocardial infarction or cardiac arrest, severe organ failure, familial Alzheimer’s Disease (AD), concomitant past and current neurological disorders (epilepsy, encephalopathy of any cause, history of severe head trauma, history of encephalitis, multiple sclerosis, Parkinson’s disease, brain tumor, and dementia due to any cause), pregnancy, prior psychiatric disorders and chronic use of neuroleptics and anticholinergic medication (depression, anxiety disorder, attention deficit hyperactivity disorder, autism spectrum disorders, posttraumatic stress disorder, schizophrenia, personality disorders, and alcohol and drug abuse), unclear IBD diagnosis, prior involvement in clinical trials, current and past history of neoplasia, use of vitamin B12 and B9 supplements, short bowel syndrome, diabetes mellitus, hypothyroidism, prior diagnosis of atrial fibrillation and uncontrolled arterial hypertension, and current use of corticotherapy.

### 2.5. Clinical Assessment

Patients included in the study provided informed consent by signing the Informed Consent Form (ICF). Following this, each patient underwent a thorough physical examination, which included measurements of blood pressure, pulse, and pulse oximetry. IBD severity was assessed using CDAI for CD patients and HBI for UC patients. A comprehensive neurological examination was then conducted. Cognitive assessments were performed using validated tools, including the Montreal Cognitive Assessment (MOCA), Memory Impairment Score (MIS), Forward and Backward Digit Span (FDS and BDS), Trail Making Test A and B (TMT-A and TMT-B), and the Digit Symbol Substitution Test (DSST). Additionally, patients completed a series of standardized questionnaires: the Depression Anxiety Stress Scales (DASS-21), the COVID-19 Impact Scale, a short cognitive assessment, and a quality-of-life questionnaire (5Q-5D-5L). Activities of Daily Living (ADL) and Instrumental Activities of Daily Living (IADL) were also performed in order to stratify patients who suffer from mild/moderate or severe cognitive impairment from patients with all-cause dementia. A comprehensive assessment of the medical history was also performed. A 5 mL blood sample was taken from the participants and stored until it was examined.

### 2.6. Laboratory Assessment

The concentrations of serum HCY, BDNF, SAA, and S100B were assessed using enzyme-linked immunosorbent assay (ELISA) kits. Each sample was tested in duplicate according to the instructions provided with the kits (Table 1).

**Figure 1:**
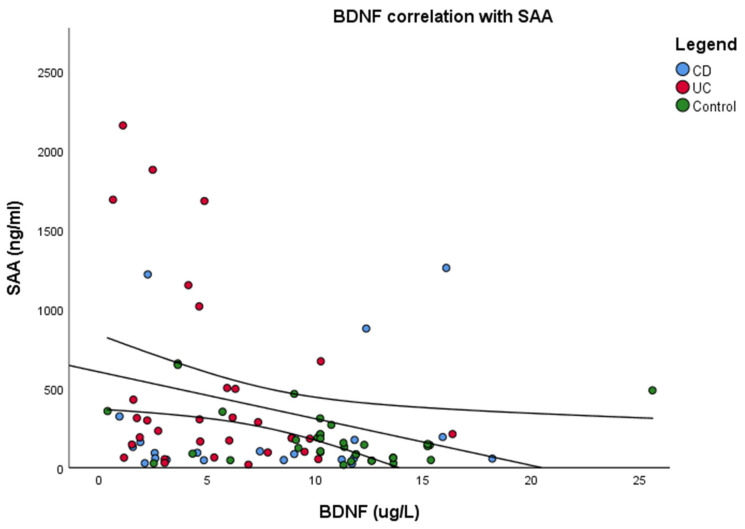
Pearson correlation Between Serum BDNF and SAA Levels in Study Participants. Scatter plot shows the correlation between serum BDNF and SAA levels. Each point represents an individual patient. The trend line illustrates the direction of correlation (R = −0.241, R^2^ = 0.058, *p* = 0.02), indicating a weak association between the two biomarkers. SAA = Serum amyloid A. BDNF = Brain derived neurotrophic factor. CD = Crohn Disease. UC = Ulcerative colitis.

For each parameter, a calibration curve was created using the protein standard provided with the kit. Absorbance was measured with a microplate reader (ClarioStar, BMGLabtech, Ortenberg, Germany), and the data were acquired and analyzed using the Mars software version 3.1 integrated into the system. A four-parameter logistic regression model was used to develop the calibration curve for quantification, and the final concentration was determined by averaging the duplicate measurements. Data are presented in Figure 1 and Table 7.

### 2.7. Statistical Analysis

Counts and percentages were used to describe categorical data. Medians and interquartile ranges (IQR) were used to describe data not following normal distribution. Comparisons between groups regarding categorical data were performed with a Chi-square test or Fisher exact test. Comparisons between three groups for data not following the normal distribution were performed with a Kruskal-Wallis test, followed by nonparametric post-hoc tests (Dunn test). To verify the associations observed in the univariate analyses between the disease and biomarkers, multivariate linear models were fit. For each model, the biomarkers were selected as dependent variables, and the disease group (CD, UC, or CG) was the exploratory variable. In case the residuals were not normally distributed, the logarithm of the dependent variable was used instead. Each model was adjusted for confounding variables, selected based on the literature and clinical or physio pathological rationale. The number of independent variables did not surpass the rule of thumb of 10 subjects per variable (degree of freedom), to prevent overfitting. For each model, the multicollinearity was checked with correlation analyses and variance inflation factor. The heteroskedasticity was checked with a Breusch-Pagan test, as well as with scale-location plots. The linear functional form was assessed with component plus residual plots. All statistical analyses were performed with the R environment for statistical computing and graphics (R Foundation for Statistical Computing, Vienna, Austria), version 4.3.2 [21].

## 3. Results

### 3.1. Study Participants

A total of 90 subjects were included in the final analysis, divided into 3 groups: 21 in the CD group, 34 in the UC group, and 35 in the CG. Demographics, such as age (*p* = 0.528), education level (*p* = 0.376), smoking index/year (*p* = 0.312), and body mass index (BMI) (*p* = 0.789), were similar across groups (Table 2). However, a higher proportion of females was observed in the CG compared to the UC and CD groups (*p* = 0.032). There was no significant difference in the proportion of participants living in rural versus urban areas (*p* = 0.22). CD and UC participants reported engaging in physical activity at least once per week with a significantly higher frequency than the CG (*p* = 0.013) (Table 2). Ten patients were excluded from the IBD groups: three due to pregnancy, five for personal reasons, and two because relocation made participation impractical, while ten subjects from the control group, out of which nine were males were excluded from the study due to non-compliance and failure to present to the 12 months visit. None of the included subjects had an impairment in the ADL and IADL scales.

Patients experiencing acute flares were recruited during hospitalization, with interviews and blood samples collected after the resolution of the flare. All patients included in the UC group had a HBI score < 2, indicating they were in clinical remission. Similarly, all patients in the CD group had a CDAI score < 150, confirming their remission status at the time of assessment. Therefore, disease activity was minimal across both groups, reducing the potential confounding effects of active inflammation on cognitive performance and biomarker levels.

The chronic treatment used by the UC and CD groups is presented in Table 3. Most of the CD and UC groups were treated with biologic therapy, encompassing 57% and 47% of the total. There were no statistically significant differences between the IBD groups.

### 3.2. Baseline Cognitive and Emotional Function in CD, UC, and the Controls: A Comparative Analysis

Baseline MoCA testing revealed significantly lower scores in the CD group compared to the CG (*p* = 0.004) and in the UC group compared to the CG (*p* = 0.017), with no significant difference between the CD and UC groups (*p* = 0.301). Similarly, MIS scores were significantly lower in the CD group (*p* = 0.022) and UC group (*p* = 0.038) compared to the CG, but no significant difference was observed between the CD and UC groups (*p* = 0.43) (Table 4). TMT-A and TMT-B completion times were higher in the CD and UC groups compared to the controls, indicating trends toward slower processing speed, attention, and executive function, though these differences were not statistically significant (*p* = 0.186 for TMT-A; *p* = 0.113 for TMT-B). DSST scores were lower in the CD and UC groups compared to the controls, suggesting potential impacts on processing speed, attention, and working memory, though not statistically significant (*p* = 0.374) (Table 4). In the FDS test, all groups showed comparable results (*p* = 0.968). However, the CD group demonstrated lower scores in the BDS test compared to the other groups, though the difference was not statistically significant (*p* = 0.6).

Patients were stratified into four cognitive categories based on their MoCA scores: Normal cognition: MoCA ≥ 26; Mild cognitive impairment (MCI): MoCA 18–25; Moderate cognitive impairment: MoCA 11–17; Severe cognitive impairment: MoCA ≤ 10. No participants showed impairments in ADL or IADL assessments, supporting the classification of cognitive impairment without concurrent functional decline. Cognitive impairment was identified in 14 (66.67%) CD patients, 16 (47.06%) UC patients, and 9 (25.71%) controls (*p* = 0.006). Moderate cognitive impairment was observed in five (23.81%) CD patients and three (8.82%) UC patients, while none of the controls exhibited such low scores (Table 5).

No statistically significant differences were observed in stress, anxiety, or depression scores between the UC, CD, and control groups (*p* > 0.5). Interestingly, psychological scores were lower in the IBD groups compared to the controls. Regarding anxiety, very severe levels were reported in three CD patients (14.29%). Mild and moderate anxiety levels were less frequent in the CD group (14.29% and 9.52%) compared to the UC group (17.65% and 14.71%) and the controls (22.86% and 17.14%). Severe depression was reported by one CD patient (4.76%) and one UC patient (2.94%), while none of the controls reported this level. Mild and moderate depression levels were less frequent in the UC group (8.82% and 8.82%) compared to the CD group (9.52% and 9.52%) and the controls (11.43% and 14.29%) (Table 6).

### 3.3. Baseline Biomarker Assessment in CD, UC, and the Controls: A Comparative Analysis

Serum biomarkers were assessed during the baseline visit. Hcy was elevated in the CD group, followed by the UC group; however, none of the changes were statistically significant (*p* = 0.369). SAA levels were highest in the UC group, followed surprisingly by the CG. Statistically significant differences were observed across all group comparisons (*p* = 0.003), while no significant difference was found between the CD group and CG (*p* = 0.561). BDNF levels were lowest in the UC group, followed by the CD group, with statistically significant differences observed across all groups (*p* < 0.001). However, no significant difference was found between the CD and UC groups (*p* = 0.071) (Table 7). S100B protein assessment did not pass the threshold value of the kit.

### 3.4. Longitudinal Findings in Cognitive Function in CD, UC, and the Controls: A Comparative Analysis

At the 1-year follow-up, small decreases in MoCA and MIS scores were observed across all groups, reflecting mild cognitive decline over time, though not statistically significant (Table 8). Notably, two CD patients who initiated anti-integrin therapy showed significant cognitive improvement, with MoCA score increases of 12 and 4 points, respectively.

At the 1-year follow-up, a small decrease in both MoCA and MIS scores was observed across all three groups (Table 9). However, none of the differences proved statistically significant (*p* > 0.05).

### 3.5. Multivariate Analysis of Biomarkers in CU, CD, and the Controls: Adjusting for Lifestyle Factors

To assess the robustness of the findings in the univariate analyses, we created multiple linear regression models predicting the biomarkers of interest based on the disease group and adjusted for age, biologic therapy, BMI, sleep hours, and practice of sport. The analysis of biomarkers revealed the following results:

○ In the univariate analyses, significant differences were noted between patient groups regarding log-transformed SAA levels (*p* = 0.003). Multivariate regression, adjusted for age, biologic therapy, BMI, sleep duration, and physical activity, revealed that UC remained significantly associated with increased log-SAA compared to the controls, whereas CD did not (*p* = 0.09, R = 0.368, R^2^ = 0.135) (Table 10).
○ Hcy: No significant differences in Hcy levels were observed between the disease groups after adjustment in the multivariate model (*p* = 0.289, R = 0.310, R^2^ = 0.096) (Table 11).
○ BDNF: Both the CD and UC groups exhibited lower BDNF levels compared to controls, with this difference maintaining significance in the multivariate analysis (*p* < 0.001, R = 0.497, R^2^ = 0.247) (Table 12).

### 3.6. Correlations Between Serum Biomarkers and Cognitive Functioning

**Figure 2:**
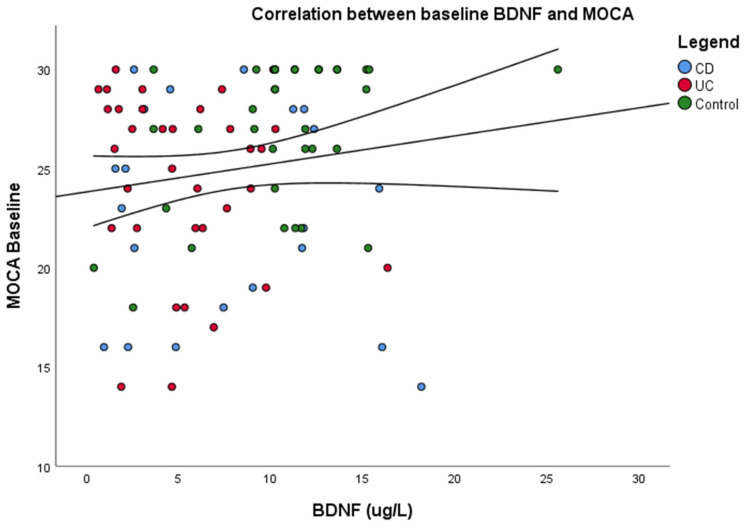
Pearson correlation Between Baseline MoCA Score and Serum BDNF Levels. Scatter plot illustrates the correlation between baseline MoCA scores and serum BDNF levels. A weak positive association was observed (R = 0.152, R^2^ = 0.023, *p* = 0.154), indicating minimal correlation between cognitive function at baseline and BDNF levels. BDNF = Brain-derived neurotrophic factor. CD = Crohn’s Disease. UC = Ulcerative colitis.

While SAA levels were elevated, BDNF levels showed a decrease, indicating an inverse correlation between inflammation and neuronal growth factor (Figure 1). Baseline MOCA scores correlated positively with BDNF levels (Figure 2).

### 3.7. Diagnostic Performance of Serum Biomarkers for Cognitive Dysfunction

**Figure 3:**
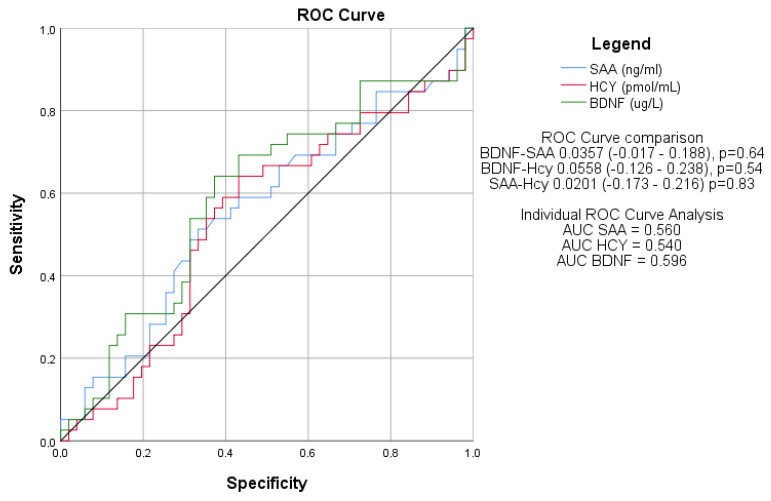
Comparison of ROC Curves for BDNF, SAA, and Hcy in Diagnosing Cognitive Dysfunction. Receiver operating characteristic (ROC) curves illustrating the diagnostic performance of serum BDNF, SAA, and Hcy in identifying cognitive dysfunction. The area under the curve (AUC) values ranged from 0.5 to 0.6, indicating limited diagnostic utility, with no significant differences between the biomarkers. SAA = Serum amyloid A. BDNF = Brain-derived neurotrophic factor. HCY = Homocysteine. CD = Crohn’s Disease. UC = Ulcerative colitis.

ROC curve analysis was performed to assess the individual diagnostic accuracy of BDNF, SAA, and Hcy in identifying cognitive dysfunction, defined by a MoCA score of less than 27 points. The area under the curve (AUC) values for all three biomarkers ranged between 0.5 and 0.6, indicating poor discriminative ability. No significant differences were observed between the AUC values of the biomarkers, suggesting that none provided a reliable diagnostic tool for cognitive dysfunction (Figure 3).

## 4. Discussions

This study provides new insights into the GBA interactions in IBD, highlighting potential links between chronic intestinal inflammation, cognitive impairment, and biomarker alterations.

Cognitive assessment using MoCA and MIS revealed that patients with IBD are more likely to exhibit MCI compared to the controls, even though deficits in daily functioning (as assessed by ADL and IADL scores) were not apparent. While MoCA and MIS scores were significantly diminished in the IBD group compared to the controls, and no significant differences were observed in TMT-A, TMT-B, or DSST, which were testing solely processing speed, attention, and executive function, it could suggest that memory is mostly affected in the patients suffering from IBD. These findings align with prior research suggesting MCI in this population [6,22,23]. However, the prevalence of cognitive impairment in UC patients (47.06%) was lower than previously reported (89.65%), likely due to differences in diagnostic criteria, study populations, and cognitive assessment tools [6]. Standardizing assessment protocols across studies would enhance comparability and provide clearer insights into cognitive outcomes in IBD populations.

The mild cognitive decline observed over one year underscores the importance of ongoing monitoring in IBD patients. Notably, cognitive improvements in two CD patients receiving anti-integrin therapy suggest a potential link between advanced treatment and cognitive outcomes, highlighting an area for future investigation.

Biomarker analyses highlighted lower BDNF levels and higher SAA levels in IBD patients, which remained significant even after adjusting for confounders. These results suggest potential links between systemic inflammation and cognitive decline in IBD populations, particularly in UC patients. Hcy levels showed no significant associations, suggesting limited relevance as a cognitive biomarker in this context. The S100B protein levels did not surpass the detection threshold of the assay kit used in this study, suggesting that its normal physiological range may be higher than the sensitivity limit of the kit [24]. This finding implies that direct neuronal damage, as indicated by elevated S100B levels, is unlikely to be the primary mechanism underlying the cognitive changes observed in our cohort. Instead, these cognitive alterations are more likely attributable to a functional impairment of the blood-brain barrier, which may disrupt neuroinflammatory homeostasis without necessarily causing extensive neuronal injury.

The independent association of elevated SAA with UC, but not CD, after adjustment for confounders, suggests differential inflammatory profiles between these disease entities. The loss of significance for CD highlights that previously observed differences may reflect confounding by other clinical or lifestyle factors rather than disease-specific inflammation alone.

The correlation between biomarkers and cognitive decline was present but weak, primarily due to the limited number of included patients, which may have affected the statistical power of the analysis. This limitation could be addressed in future studies by increasing the sample size, ensuring a more comprehensive patient inclusion strategy, and incorporating additional biomarkers or longitudinal assessments to strengthen the analysis of cognitive decline in IBD patients. ROC curve analysis indicated low performance in diagnostic cognitive impairment, but further studies involving a larger number of patients could shape this pattern and increase the sensitivity and specificity of this biomarker set.

Contrary to the existing literature, stress, anxiety, and depression scores were lower in IBD patients compared to the controls [23,25,26,27]. This unexpected finding may reflect effective coping mechanisms, social support, or selection bias in the study cohort. However, patients were required to be in a clinical remission period, which could also positively influence their depression, anxiety, and stress scores. Also, biologic treatment could improve their affective lifestyle. Further research is needed to explore the psychosocial and environmental factors influencing psychological resilience in IBD populations.

One of the main limitations of this study was the widespread fear of hospitals, a phenomenon exacerbated by the COVID-19 pandemic. However, this challenge was mitigated by the fact that many patients required regular medical prescriptions at monthly or bi-monthly intervals, ensuring continued participation. Language barriers also posed a challenge, as several cognitive assessment questionnaires have not been clinically validated in Romania, restricting the selection of available tools. Additionally, telephonic and video-based cognitive testing were not feasible due to the absence of officially approved remote testing methods. Lastly, the relatively small sample size and the limited follow-up period represent further limitations, potentially restricting the generalizability of the findings. Biomarkers, such as C-reactive protein and fecal calprotectin, were unavailable for many patients due to the nature of their consultations; clinical assessment tools were utilized to distinguish between remission and active disease. Specifically, the CDAI and the HBI were employed to stratify patients and ensure accurate classification. From the initial number of patients, a total of 20 were lost to follow-up due to different reasons. Since seven of the CD patients were lost and only three from the UC group, it created a gap in the total number of IBD patients. Also, in the control group, out of 10 lost to follow-up patients, 9 were males, thus creating a significant difference in gender between the 3 groups. Some of the serum BDNF is not produced by the brain but by platelets and peripheral tissues, and it might be modified by certain medications, such as corticotherapy.

Future research should focus on larger, longitudinal studies involving diverse populations to confirm observed trends in cognitive performance and biomarker levels among IBD patients. Such studies should assess the influence of disease severity, inflammation, and treatment on cognitive and psychological outcomes, while also identifying protective factors and effective interventions.

## 5. Conclusions

This study provides valuable insights into cognitive and biomarker differences in IBD patients, emphasizing the need for further investigation. While cognitive impairments in IBD are detectable, they are not universally severe and may occur without significant functional deficits. Lower BDNF and higher SAA levels suggest a potential link between systemic inflammation and cognitive decline in IBD, particularly in UC patients. Future research should prioritize longitudinal studies, standardized cognitive assessment protocols, and additional biomarker exploration to clarify disease-related cognitive changes and inform targeted interventions.

## Figures and Tables

**Table 1:** Specifications and Sources of ELISA Kits for Biomarker Measurement (CV = coefficient of variation).

Biomarker	ELISA Kit	Kit Performance
HCY	AssayGenie, Dublin, Ireland, HUFI04768	Detection range = 7.813–500 pmol/mL; Sensitivity < 4.688 pmol/mL Intra-assay precision CV < 8%; Inter-assay precision CV < 10%
BDNF	ABclonal, Woburn, MA, USA, RK00074	Detection range = 23.4–1500 pg/mL Sensitivity < 6.3 pg/mL Intra-assay precision CV < 10%; Inter-assay precision CV < 15%
SAA	ABclonal, Woburn, MA, USA, RK04228	Detection range = 0.156–10 ng/mL; Sensitivity < 0.071 ng/mL Intra-assay precision CV ≤ 10%; Inter-assay precision CV ≤ 15%
S100B	ABclonal, Woburn, MA, USA, RK02234	Detection range = 46.9–3000 pg/mL; Sensitivity < 23.5 pg/mL Intra-assay precision CV ≤ 10%; Inter-assay precision CV ≤ 15%)

**Table 2:** Comparison of Demographics, Clinical Assessments, Cognitive Scores, and biomarkers Among the Three Groups (IQR = interquartile range; CI = confidence interval).

Group	CD Group (*n* = 21)	UC Group (*n* = 34)	CG (*n* = 35)	*p* (CD-UC, CD-CG, UC-CG)
Age (years), median (IQR)	39 (29–49)	44.5 (32.75–60.75)	39 (32.5–59)	0.528 (0.724, 0.568, 0.665)
Education (years), median (IQR)	15 (12–16)	15 (12–16)	15 (12–18)	0.376 (0.759, 0.615, 0.347)
Smoking index/year, median (IQR)	0 (0–6)	0 (0–0)	0 (0–0)	0.312 (0.355, 0.39, 0.553)
Body-mass index (BMI) (kg/m^2^), median (IQR)	24.4 (22.6–26.8)	24.85 (23.13–27.95)	23.9 (21.45–27.95)	0.789 (0.896, 0.829, 1)
Gender (Female), *n* (%)	10 (47.62)	14 (41.18)	25 (71.43)	**0.032** (0.64, **0.07, 0.01)**
Living environment (Rural), *n* (%)	2 (9.52)	9 (26.47)	5 (14.29)	0.22 (0.12, 0.6, 0.2)
Physical activity (No), *n* (%)	15 (71.43)	17 (50)	29 (82.86)	**0.013 (0.11**, 0.31, **0.001**)
ADL, median (IQR)	6 (6)	6 (6)	6 (6)	1 (1, 1, 1)
IADL, median (IQR)	8 (8)	8 (8)	8 (8)	1 (1, 1, 1)

**Table 3:** Comparison of medication of CD and UC patients.

Treatment	CD (*n* = 21)	UC (*n* = 34)	*p*
Standard therapy, *n* (%)	8 (38%)	15 (44.1%)	0.42
Biologic, *n* (%)	12 (57%)	16 (47%)	0.2
None, *n* (%)	1 (4.7%)	3 (8.8%)	0.63

**Table 4:** Comparison of Cognitive Scores Among the Three Groups (IQR = interquartile range; CI = confidence interval; MOCA = Montreal Cognitive Assessment; MIS = Memory Impairment Scale, FDS = Forward Digit Span; BDS = Backward Digit Span; DSST = Digit Symbol Substitution Test).

Group	CD Group (*n* = 21)	UC Group (*n* = 34)	CG (*n* =35)	*p* (CD-UC, CD-CG, UC-CG)
MOCA test-baseline, median (IQR)	23 (18–28)	26 (22–28)	28 (25–30)	**0.003** (0.301, **0.004**, **0.017**)
MIS-baseline, median (IQR)	12 (8–14)	13 (9.25–14)	14 (12.5–15)	**0.015** (0.43, **0.022**, **0.038**)
FDS, median (IQR)	10 (9–12)	10 (8–12)	10 (9.5–11)	0.968 (1, 0.855, 1)
BDS, median (IQR)	5 (4–8)	6 (4–6.75)	6 (5–8)	0.6 (0.822, 1, 0.549)
Trail making A (seconds), median (IQR)	43 (33–59.5)	38.85 (31.05–59)	35 (26–42)	0.186 (0.443, 0.215, 0.31)
Trail making B (seconds), median (IQR)	107 (72–155)	100 (54.33–154.25)	72 (65–80.5)	0.113 (0.269, 0.106, 0.339)
DSST, median (IQR)	43 (28.5–48.5)	40 (33–48.25)	46 (39–51)	0.374 (0.658, 0.186, 0.322)

**Table 5:** Cognitive Categories Comparison Among the Three Groups.

Group	CD (*n* = 21)	UC (*n* = 34)	CG (*n* = 35)	*p*-Value (CD-UC, CD-CG, UC-CG)
Cognitive dysfunction (total), *n* (%)	14 (66.67)	16 (47.06)	9 (25.71)	**0.006** (0.15, **0.003**, 0.06)
Mild cognitive impairment, *n* (%)	9 (42.86)	13 (38.24)	9 (25.71)	0.35 (0.73, 0.18, 0.26)
Moderate cognitive impairment, *n* (%)	5 (23.81)	3 (8.82)	0 (0)	**0.01** (0.23, **0005**, 0.11)
Normal, *n* (%)	7 (33.33)	18 (52.94)	26 (74.29)	**0.01** (0.15, **0.003**, 0.06)

**Table 6:** Comparison of Psychological Factors and Activities of Daily Living Among the Three Groups (IQR = interquartile range; CI = confidence interval).

Group	CD Group (*n* = 21)	UC Group (*n* = 34)	CG (*n* = 35)	*p* (CD-UC, CD-CG, UC-CG)
Stress score, median (IQR)	6 (4–10)	6 (2–13.5)	8 (4–13)	0.639 (0.884, 0.608, 1)
Anxiety score, median (IQR)	6 (0–8)	6 (0–8)	8 (2–10)	0.541 (0.834, 0.588, 0.821)
Depression score, median (IQR)	2 (0–8)	2 (0–6)	4 (0–8)	0.813 (0.716, 1, 1)

**Table 7:** Comparison of biomarkers Among the Three Groups (IQR = interquartile range; CI = confidence interval).

**Group**	**CD Group** **(*n* = 21)**	**UC Group** **(*n* = 34)**	**CG** **(*n* = 35)**	***p* (CD-UC, CD-CG, UC-CG)**
SAA (ng/mL), median (IQR)	92 (51–175)	259.5 (150.75–629)	136 (62.5–210)	**0.003** (**0.009**, 0.561, **0.007**)
Hcy (pmol/mL), median (IQR)	2802 (2028–5311)	2500.5 (1639.5–6045.5)	2287 (2012–3172.5)	0.369 (0.587, 0.501, 0.443)
BDNF (pg/mL), median (IQR)	7437 (2570–11,785)	4771 (2289.25–7552.25)	10,735 (9160.5–12,602.5)	**<0.001** (0.071, **0.035**, **<0.001**)

**Table 8:** Comparison of Cognitive Scores at 1 year visit (IQR = interquartile range; CI = confidence interval).

Group	CD Group (*n* = 21)	UC Group (*n* = 34)	CG (*n* = 35)	*p* (CD-UC, CD-CG, UC-CG)
MOCA- 1 year visit, median (IQR)	23 (18–27)	25.5 (21–28)	27 (23.5–30)	**0.012** (0.388, **0.016**, **0.035**)
MIS- 1 year visit, median (IQR)	10 (7–13)	12 (9–14)	14 (11–15)	**0.033** (0.67, 0.069, **0.041)**

**Table 9:** Comparison of MOCA and MIS score at 1-year follow-up.

Group	MOCA Test- Baseline, Median (IQR)	MOCA- 1 Year Visit, Median (IQR)	*p*	MIS- Baseline, Median (IQR)	MIS- 1 Year Visit, Median (IQR)	*p*
CD Group (*n* = 21)	23 (18–28)	23 (18–27)	0.9681	12 (8–14)	10 (7–13)	0.764
UC Group (*n* = 34)	26 (22–28)	25.5 (21–28)	0.741	13 (9.25–14)	12 (9–14)	0.483
CG (*n* = 35)	28 (25–30)	27 (23.5–30)	0.771	14 (12.5–15)	14 (11–15)	0.440

**Table 10:** Multiple linear regression predicting the logarithm of SAA based on disease, and adjusted for age, biologic therapy, body mass index, number of sleep hours, and practice of sport.

Characteristic	B Adjusted	(95% CI)	*p* Value
Disease (CD vs. CG)	−0.041	(−0.10–0.96)	0.916
Disease (UC vs. CG)	0.932	(0.29–1.57)	**0.005**
Age	0.001	(−0.016–0.018)	0.889
Biologic therapy	−0.012	(−0.665–0.641)	0.971
BMI (kg/m^2^)	−0.014	(−0.06–0.038)	0.579
Sleep hours/24 h	0.03	(−0.255–0.333)	0.811
Physical activity	−0.23	(−0.79–0.343)	0.433

**Table 11:** Multiple linear regression predicting the logarithm of Hcy based on disease, and adjusted for age, biologic therapy, body mass index, number of sleep hours, and practice of sport.

Characteristic	B Adjusted	(95% CI)	*p*
Disease (CD vs. CG)	0.43	(−0.394–0.481)	0.113
Disease (UC vs. CG)	−0.077	(−0.446–0.291)	0.741
Age	0.001	(−0.009–0.011)	0.812
Biologic therapy	0.331	(−0.045–0.707)	0.084
BMI (kg/m^2^)	0.019	(−0.011–0.049)	0.215
Sleep hours/24 h	−0.08	(−0.249–0.09)	0.352
Physical activity	−0.107	(−0.434–0.221)	0.519

**Table 12:** Multiple linear regression predicting the logarithm of BDNF based on disease, and adjusted for age, biologic therapy, body mass index, number of sleep hours, and practice of sport.

Characteristic	B Adjusted	(95% CI)	*p*
Disease (CD vs. CG)	−0.631	(−1.13–−0.125)	**0.015**
Disease (UC vs. CG)	−0.897	(−1.32–−0.471)	**<0.001**
Age	0.003	(−0.009–0.014)	0.642
Biologic therapy	0.311	(−0.123–0.746)	0.158
BMI (kg/m^2^)	0.03	(0.005–0.064)	0.092
Sleep hours/24 h	−0.085	(−0.28–0.111)	0.391
Physical activity	−0.144	(−0.523–0.235)	0.451

## Data Availability

Study data is available upon reasonable request from the corresponding author. The data will not be published on a public platform for ethical reasons. The pseudonymization of the participants will be removed from the database if a request for access is received.

## Abbreviations

AD: Alzheimer’s disease; ADL: activities of daily living; BDNF: brain-derived neurotrophic factor; BDST: backward digit span testing; CD: Crohn’s disease; CDAI: Crohn Disease Activity Index; CG: Control group; CNS: Central Nervous system; CV = Coefficient of variation; DASS: Depression Anxiety Stress Scale; DSST: Digit Symbol Substitution Test; ELISA: enzyme-linked immunosorbent assay; ENS: Enteric Nervous system; FDST: forward digit span testing; GBA: gut–brain axis; HBI: Harvey-Bradshaw Index; Hcy: homocysteine; IADL: Instrumental Activities of Daily Living; IBD: inflammatory bowel disease; IQR = Interquartile range; MCI: mild cognitive impairment; MIS: memory impairment score; MOCA: Montreal Cognitive Assessment; S100B: S100 calcium-binding protein B; SAA: serum amyloid A; SCCAI: Simple Clinical Colitis Activity Index; TMT: Trail Making Test; UC: ulcerative colitis.
